# Supplementary material for: Cobalt ion interaction with TMEM16A calcium-activated chloride channel: Inhibition and potentiation
Source: PLoS One. 2020 Apr 17;15(4):e0231812. doi: 10.1371/journal.pone.0231812 (PMC7164836; doi:10.1371/journal.pone.0231812)
Supplement: Attachment — Submitted filename: Response to Reviewers.docx [file pone.0231812.s002.docx]

**Response to Reviewers**

We thank reviewers for their helpful comments/criticisms in improving our manuscript. The response to reviewers’ criticisms are as follows, where the reviewers’ comments are colored in black while the authors’ responses are colored in red. Besides the manuscript, a “mark-up copy of the manuscript is also provided in which the changes made to the original version are colored in red.

Reviewer #1: The submitted manuscript presents an in-dept investigation on the ion conductivity changes in a membrane Ca-activated chloride channel upon competitive binding of Co2+ ions. This protein is a transmembrane homodimer, each subunit having two high affinity binding sites for calcium. Correspondingly, the interpretation of just the current recordings upon the displacement of one type of metal by another type of metal is challenging. The authors have accumulated a respectable volume of current recordings in diverse conditions (including 10 mutant proteins). It would have definitely helped if the stability constants for cobalt ions were measured for all possible binding sites (and in the mutants). However, the complexity of lipid environment adds a whole extra layer of uncertainty. So, it is understandable that the data, as presented, do not make a completed picture.

Long story short, there are a number of questionable parts in the authors’ treatment of data, plus the English of the article is very heterogeneous. All that let me only to recommend a major revision of the manuscript. I have compiled the specific comments below.

Major comments
1. Ln. 101, 104: “…alter the affinity of Ca2+ in activating the channel…” — the affinity and channel activation should be discussed separately.

Response: We agree that separating the binding affinity from the channel activation would result in a better distinction among different mechanistic steps in the operation of ligand gated channels. Nonetheless, the binding affinity and the activation of ligand-gated channels are tightly coupled, and thus are difficult to separate. In line 101-104 (line 99-102 in the revised version), we simply refer to other’s work showing the functional evidence for several acidic residues in forming the Ca^2+^ binding sites (later confirmed by the high-resolution channel structures). The description here does not intend to discuss the mechanisms of binding affinity and channel activation. To ease the reviewer’s concern, and to emphasize a phenomenological description, we now replace the word “affinity” by “half-effective concentration.”

2. Ln. 169: “…the concentration of NaCl in the intracellular solution was reduced according to the extra [Cl-] from the added [CoCl2].” — such adjustments would have altered the ionic strength of the solution. Few words should be added on the possible impact on the observations.

Response: The suggestion from the reviewer is well taken. We now add a sentence in METHOD that in the extremely high concentration of Co^2+^ (such as 5 – 20 mM), the ionic strength of the solution may have an impact on the effect. Thus, “the effects obtained with high divalent cation concentrations (such as 5 - 20 mM) may be less accurate.” (line 172-174).

3. “Data analysis” section is very poorly written, hard to read (compared e.g. to the Discussion, which is good).

Response: We thank the reviewer for this comment. We now extensively revise the section of “Data analysis.” (line 206-228).

4. Ln. 209: “was fit to a single-exponential function” — What kind of function is this? Apparently, it is valid only for the chosen concentration of EGTA?

Response: The single-exponential equation is now explicitly given in METHOD (line 222). The parameter τ_off_ refers to the time constant of the current-decreasing process upon removing the activation ligand Ca^2+^ (at the end of the recording traces as those shown in Fig. 2 B). The wash solution contains no Ca^2+^ (but with EGTA). Therefore, the value of τ_off_ should practically not depend on the concentration of EGTA in the wash solution provided the EGTA concentration is high enough to chelate most Ca^2+^ ions so that the wash solution has “zero” [Ca^2+^]. All experiments were conducted with the same wash solution, containing 0.1 mM EGTA, while the contaminating [Ca^2+^], as shown in Suppl Fig. 1, is less than 1 µM.

5. The selection of a Langmuir-type equation for the data analysis is highly questionable. Why not employ standard competitive binding models?

Response: We use the Langmuir-type equation to simply report the half-effective concentration of Co^2+^ in inhibiting TMEM16A current induced by various [Ca^2+^]_i_. The analysis is meant to qualitatively show that as [Ca^2+^]_i_ is increased, the half-effective concentration of Co^2+^ is also increased. This observation then suggests to us that the inhibition may result from the competition of Co^2+^ and Ca^2+^ for the channel activation sites similar to our previous experiments of the Mg^2+^ inhibition of TMEM16A. We have also presented two other types of experiments (in Fig. 4 & 5, separately) to strengthen the conclusion that Ca^2+^ and Co^2+^ compete for the channel activation sites.

By looking at the data in Table 1, however, we feel a simple standard competitive model may not quantitatively describe the data well. For example, in Table 1 one can see that as the [Ca^2+^]_i_ used to induce TMEM16A current is increased, the increase of the half-effective Co^2+^ concentration for inhibiting the current is not linearly correlated with the increase of [Ca^2+^]_i_. This is likely due to a difference in the state-dependent (open vs closed state) binding to the channel activation sites between Ca^2+^ and Co^2+^. To illustrate this point quantitatively, however, a more sophisticate competition model is required, a task beyond the scope of this paper. Because there was no report on the effects of Co^2+^ on TMEM16A channel, we report both the inhibition and the potentiation phenomena in this paper, and the aim of the paper is to characterize the inhibition and the potentiating effects without going too deep into modeling the results. An in-depth modeling of just the Co^2+^ inhibition data may become distractive for the flow of the paper.

6. Ln. 321-323, 340-341: “the correlation of Co2+ inhibition with the sidechain hydrophobicity is weak (Fig 7 A & B, middle panel). Inhibition appears to correlate with the molecular volume of the amino acid placed at position 589…” — I don’t see any correlations in these cases.

Response: We did mention that the correlation of Co^2+^ inhibition with the molecular volume of the amino acid placed at position 589 is much weaker than the correlation of the Co^2+^ inhibition with τ_off_. The degree of correlation is perhaps a bit subjective. We now state the correlations of the inhibition and potentiation with those parameters following the comments of the reviewer.(line 327-329 & line 344-346).

7. Fig. 8: The inverse correlation between the mutant residue volume and the current, could it be interpreted as a mechanical obstruction of the channel?

Response: Mechanical obstruction is of course one possibility. However, our data shows that the potentiation does not perfectly correlate with the sidechain volume. For example, tryptophan appears to be an outlier. Its sidechain is much larger than that of phenylalanine or tyrosine but the potentiation of Y589W is larger than that observed in WT (Y589) or Y589F. Thus, interpreting these data by mechanical obstruction of the pore will not be optimal.

Minor and technical comments

1. Throughout (e.g. ln. 32, “…charged sidechain carboxylates to coordinate…”): ‘carboxylate’ is an anion of a carboxylic acid; the group is carboxyl (or ‘ionized carboxyl’ if one prefers).

Response: We consider that the word “carboxylate” describes the anionic carboxyl group just fine because “carboxylate” is extensively used in most biochemistry textbooks to refer to the negatively-charged carboxyl group in the sidechain of glutamate or aspartate. Reviewer #2 apparently also used the word “carboxylate” to refer to the negatively charged carboxyl group (see Reviewer # 2’s Major points #2). We specifically use the word carboxylate to differentiate with the protonated carboxyl group because the latter does not coordinate Ca^2+^ well as shown in a single E to Q point mutation out of these Ca^2+^ coordinating residues where the Ca^2+^ apparent affinity in activating the channel is reduced by several-hundred fold.

2. Ln. 41: “…mutating a pore residue…” — in the abstract it’s preferable to be specific about which residue was mutated.

Response: Correction is made according to the reviewer’s suggestion (in line 40)

3. Ln. 64: “…conduction of ionic currents across lipid membranes. The physiological roles of the current conduction…” — it’s either ‘ion current’ or ‘ionic conduction’.

Response: Correction is made according to the reviewer’s suggestion (line 63-64).

4. Ln. 68: “…are dimeric proteins consisting of two identical subunits…” — a proper term is ‘homodimeric’.

Response: Correction is made according to reviewer’s suggestion (line 68).

5. Ln. 96: “…each protein subunit consists of a set of Ca2+-binding sites…” — it doesn’t consist, but has exactly two binding sites, as follows from the published structures.

Response: The sentence is revised according to reviewer’s suggestion (line 95).

6. Ln. 106 : “…the binding alters the rectification of the Cl- flux through the channel pore by reducing the negative charge from these Ca2+-coordinating residues…” — not clear, should be rephrased.

Response: The sentence is rephrased (line 105-107)

7. Ln. 210: Fig 5C is cited not in sequence (the previous one was Fig 2B).

Response: Revision is made in the relevant paragraph (line 206- line 211).

8. Ln. 514: What is CLC-0?

Response: CLC-0 is a Cl- channel from *Torpedo* electric organ. We now replace “CLC-0” by “*Torpedo* CLC-0 Cl- channel” (line 520).

9. Ln. 521: Should be “vestibule”.

Response: The typo is now corrected (line 527). Thanks.

Reviewer #2: TMEM16A is a Ca2+-activated Cl- channel that plays key roles in diverse physiological processes including transepithelial Cl- transport. TMEM16A functional studies have been instrumental in deciphering the structure-function of the channel. For example, studies have shown that plotting Ca2+ activation of TMEM16A currents requires a Hill coefficient greater than 1, thereby consistent with multiple Ca2+ binding to activate TMEM16A currents. Moreover, functional studies demonstrated that the channel has two independent Cl- conducting pores.

Manuscript synopsis: Here, the authors explored TMEM16A gating using the divalent Co2+. Nguyen et al. report that Co2+ is a competitive inhibitor for the Ca2+ activation of TMEM16A. They also found that at high concentrations, Co2+ can potentiate Ca2+-evoked Cl- currents. These results were supported by inside-out patch experiments using intracellular Ca2+ application to activate TMEM16A, and application Co2+ to inhibit or potentiate these Ca2+-evoked Cl- currents. By varying concentrations of Ca2+ and Co2+ that were applied, the authors further characterize how Ca2+ and Co2+ regulate TMEM16A conducted Cl- currents. Additional experiments include mutations of the pore residue Y589 which were used to describe the relationship of Co2+ inhibition to amino hydrophobicity, volume, as well as the deactivation kinetics of the TMEM16A channel.

The experiments presented in this manuscript clearly demonstrate inhibition and potentiation Co2+ on TMEM16A Ca2+-evoked Cl- currents. Additionally, these experiments showing the effect of Co2+ on TMEM16A Ca2+-evoked Cl- current are well described and easy to understand. However, this is not as true for the Y589, whose interpretation in the manuscript somewhat confusing. The data included in this manuscript are interesting and should be published. However, improving the writing – specifically motivating the experiments presented in figures 6-10 and discussing the interpretation of these data - will create a more impactful manuscript.

Major points:
1) The motivation for the experiments depicted in figures 6-10 is not easily accessible to the reader. Even though understanding the characteristics of TMEM16A itself is important for the field, the specific angle for why we need to learn more about the biphasic dose response of TMEM16A is not stated. Moreover, reasons why the Y589 mutant was chosen for these experiments, or why this residue was targeted for multiple mutations, even why the amino acids used for these mutations were chosen should be clarified. Perhaps addressing how these data fit with current understanding of TMEM16A gating, or will direct future experimental directions will help the reader.

Response: The experiments depicted in figure 6-10 address the characteristics of the Co^2+^ potentiation effect on TMEM16A, which was in fact an serendipity finding (we started this research by expecting a Co^2+^ inhibition effect only). The choice of Y589 mutation was because of the finding in the literature that Y589A has a higher apparent Ca^2+^ affinity in activating the channel. Thus, we study this mutant with an expectation that the Co^2+^ inhibition effect will be weaker in this mutant. Nonetheless, when examining the effect of Co^2+^ on the WT channel and on the Y589A mutant, a potentiation effect just jumped out. Furthermore, we did not know in advance that the sidechain volume of the introduced residue has some correlation with the potentiation effect before constructing multiple mutants of Y589 for the Co^2+^ inhibition experiments. We agree that the motivation for those potentiation experiments was not present at the beginning of the experiments, but this is just the way the research process evolved. To reflect this fact, we now more clearly indicate that the potentiation effects of Co^2+^ are serendipity findings (now in line 125 and line 340-343).

2) The manuscript alludes to the importance of amino acid carboxylates (line 97) in coordinating Ca2+ but does not address this concept in the context of the Co2+ findings and the Y589 mutants. The importance of these Y589 experiments in addressing the biphasic dose-response is also not clear. Perhaps further discussion on how these mutants address the main question could help.

Response: We have stated in INTRODUCTION (line 95-96) that the carboxylates of the amino acids in the Ca^2+^-activation sites (which do not include residue 589) are important for binding divalent cations. Since we conclude that Co^2+^ is a competitive inhibitor for Ca^2+^ activation, these multiple carboxylate groups should also be responsible for interacting with Co^2+^. We now explicitly state this conclusion in DISCUSSION (line 454-455).

Regarding the amino acid sidechain carboxylates in the context of Y589 mutants that affect the Co^2+^ potentiation (and thus biphasic Ca^2+^ dose-response), we have discussed (in DISCUSSION, line 499-500 of the revised manuscript) that the sidechain of the residue at position 589 likely DOES NOT directly interact with the divalent cations that generate the potentiation effect. We have instead offered a mechanism that the potentiation of TMEM16A current by high concentrations of divalent cations likely results from divalent cation binding to phospholipids in the pore region (line 510-513). This suggestion was based on two observations: (1) the observation of a cleft in the pore wall of TMEM16 molecules as seen in the high-resolution structures; (2) the observation in this paper that the larger the sidechain volume of the residue at position 589, the smaller the degree of potentiation. We suggested that this latter observation likely reflects a possibility that the larger the sidechain volume of a pore residue at position 589, the less phospholipids can be accommodated in the pore (line 526-528). We have also discussed the similarity between the Co^2+^-induced current potentiation and the Ca^2+^-induced potentiation (and thus the biphasic dose-response curve) (the entire 4^th^ paragraph of DISCUSSION). Thus, the biphasic dose-response curve of TMEM16A results from two separate effects of Ca^2+^: a channel activation (a gating effect) by high-affinity (in µM) Ca^2+^ binding to the activation sites, and a current potentiation through the opened pore (a permeation effect) by low affinity (in mM) Ca^2+^ binding.

3) Lines 71-84 and Figure 1 highlight TMEM16F and its structural properties. It is not clear how this information adds to or sets up the motivation for the question being addressed in this manuscript.

Response: The structure of TMEM16F is similar to that of TMEM16A not just in the overall structural architecture but in the scenario that phospholipids may contribute to lining the wall of the ion-transport pathway. The presence of phospholipids in the ion-transport pathway of TMEM16 molecules is probably a consensus in the field suggested from the observed open cleft in the ion-transport pathway (where the phospholipids may reside), as shown in Fig. 1. The functional role of phospholipids in TMEM16F transport pathway is clear because this molecule is a phospholipid scramblase. However, the functional role of phospholipids in TMEM16A, a classical ligand-gated channel, is less clear. This paper presents evidence that Co^2+^ (and Ca^2+^) potentiation like results from a low-affinity binding of divalent to phospholipids near the intracellular pore entrance. Although this paper deals with TMEM16A only, we think it is important to remind the readers that TMEM16F also has phospholipids in the ion-transport pathways.


Minor points:
1) Lines 42-43 refer to Ca2+ as potentiating TMEM16A and not activating. Are the data suggesting that Ca2+ plays two roles?

Response: Yes. A low concentration (µM) of Ca^2+^ activates (opens) TMEM16A while a high concentration (mM) of Ca^2+^ potentiates the Cl^-^ current through the open pore of TMEM16A.

2) Correlations described in Figures 7 and 8 are not apparent expect for τoff. Are there patterns or features of the selected amino acids that should be described to help with the interpretation? The data are difficult to appreciate as described currently. Additionally, how these data relate to the overall question is quite unclear.

Response: The correlation of Co^2+^ inhibition (Fig. 7) with τ_off_ is the most apparent one while the sidechain volume of the introduced amino acid at position 589 has the best correlation with the Co^2+^ potentiation (Fig. 8). We have offered the rationale for both correlations. In Fig. 7, since Ca^2+^ and Co^2+^ compete for the same sites, the slower the Ca^2+^ unbinding (large τ_off_), the less the Co^2+^ inhibition. This phenomenon in fact provides further argument that Co^2+^ inhibition indeed results from the competition of Co^2+^ and Ca^2+^ for the channel activation sites. Regarding the correlation between the degree of potentiation with the sidechain volume of the residue at position 589 (Fig. 8), we suggested that the sidechain does not directly interact with the potentiating divalent cations. Rather, the sidechain volume of a residue at position 589 (which is in the pore) may affect the number of phospholipid molecules that can be accommodated in the pore region. The potentiating divalent cations bind to the phospholipid head group, and reduce (or screen) the negative charge from the lipid head group in the pore region. The smaller the sidechain of the residue, the more phospholipids in the pore, and therefore the larger the potentiation effect. We have discussed these points in the DISCUSSION.

3) The manuscript is written as though it is a follow up to another study in a way that makes the current form of this one difficult to grasp and appreciate. The data are interesting and present new information about TMEM16A, and providing more rationale for the experiments conducted will enhance the value of the data.

Response: We thank the reviewer for this comment. The inhibition of TMEM16A by Co^2+^ was indeed motivated by our previous study of Mg^2+^ inhibition on the TMEM16A channel. The Co^2+^ effect in potentiating the TMEM16A current is a serendipity finding as explained in the response to the major point #1. In any case, we have revised the writing of the manuscript and have provided more rationale for the experiments as suggested by the reviewer.
